# Carcinoma of Unknown Primary: Integrating Timely Interventions and Molecular Advancements for Improved Outcomes

**DOI:** 10.7759/cureus.76035

**Published:** 2024-12-19

**Authors:** Supriya Peshin, Mamtha Balla, Laura Tordjman, Nagaishwarya Moka

**Affiliations:** 1 Internal Medicine, Norton Community Hospital, Norton, USA; 2 Internal Medicine, MD Anderson Cancer Center, Houston, USA; 3 Radiology, RP Bluegrass Diagnostic Radiology, Somerset, USA; 4 Hematology and Medical Oncology, University of Kentucky College of Medicine, Lexington, USA

**Keywords:** afp, ca 19-9, carcinoembryonic antigen (cea), carcinoma of unknown primary (cup), her2 amplification

## Abstract

Carcinoma of unknown primary (CUP) is a diverse group of malignancies characterized by metastatic disease without an identified primary site. It typically presents with a poor prognosis due to widespread metastasis at diagnosis. This report discusses a 58-year-old female patient with advanced CUP and diffuse liver metastasis. Initially misdiagnosed with sciatica, her persistent upper abdominal pain led to further imaging, which revealed multifocal liver lesions, retroperitoneal lymphadenopathy, and small pulmonary nodules.

While identifying the primary tumor, tumor markers indicated elevated alpha-fetoprotein (AFP) and carbohydrate antigen 19-9 (CA 19-9) with normal carcinoembryonic antigen (CEA). The initial liver biopsy revealed benign tissue, prompting a second biopsy due to the high index of suspicion for carcinoma based on imaging findings. Immunohistochemistry revealed poorly differentiated carcinoma positive for HER2 (3+) and other markers, suggesting HER2-directed therapy as a treatment option. Given her frailty and tumor burden, a multidisciplinary team recommended chemotherapy with carboplatin and gemcitabine, alongside supportive care measures.

This case underscores the complexity of CUP workup and management, where a patient’s clinical stability may necessitate prompt treatment over exhaustive diagnostics. Given its potential benefits, the decision to integrate next-generation sequencing (NGS) as part of the workup and therapy highlights the role of tailored treatment in CUP management. Collaborative, multidisciplinary approaches are crucial for developing effective treatment plans and providing optimal patient outcomes. Ongoing research is essential to enhance the understanding and treatment of CUP, ensuring continued adaptation to advancing therapeutic strategies in clinical practice.

## Introduction

Carcinoma of unknown primary (CUP) is a heterogeneous group of cancers where the primary origin remains unidentified despite comprehensive diagnostic evaluation. CUP accounts for 3%-5% of all malignancies and is often associated with a poor prognosis due to poor differentiation, aggressive presentation, and widespread metastases at diagnosis [1,2]. The management of CUP can be challenging due to the lack of a primary tumor site, which complicates tailored treatment decisions [3,4]. This case report presents the complex diagnostic and therapeutic challenges in managing a 58-year-old female patient with advanced CUP, highlighting the role of next-generation sequencing (NGS), which revealed HER2 positivity.

## Case presentation

A 58-year-old female patient presented with a complex medical history significant for several risk factors and comorbidities. She had a 60-pack-year smoking history and prior heavy alcohol consumption, which ceased four years ago. Her medical history included anxiety, depression, hypercholesterolemia, acute hepatitis, and a prior basal cell carcinoma resection. Her family history was notable for malignancies, including colon and prostate cancers on her father’s side.

Initial presentation and diagnostic workup

**Figure 1:**
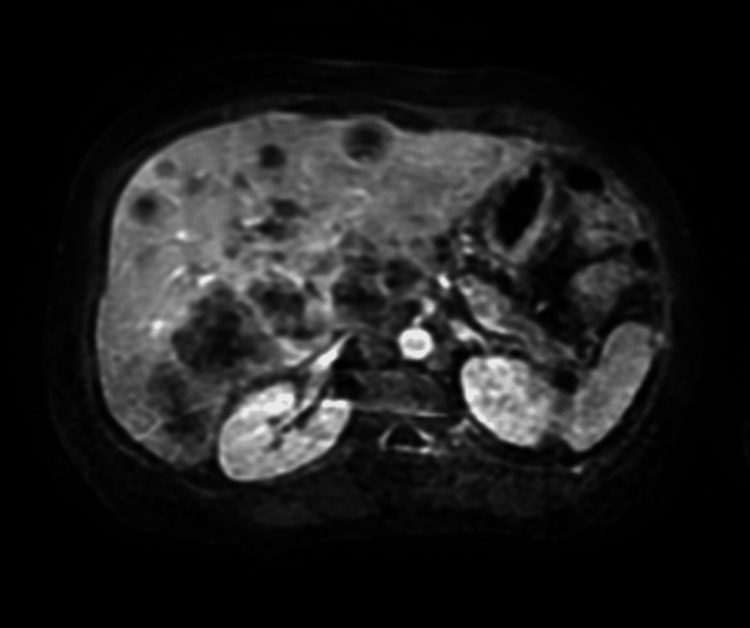
CT scan showing multifocal liver lesions, retroperitoneal lymphadenopathy, omental caking, mild pelvic ascites, and two small pulmonary nodules (2 mm and 3 mm).

In July 2024, the patient reported upper abdominal pain radiating to her hip, initially attributed to sciatica, and managed conservatively with physical therapy. However, the persistence of symptoms led to further evaluation in August 2024. Imaging studies revealed multifocal liver lesions, retroperitoneal lymphadenopathy, omental caking, mild pelvic ascites, and two small pulmonary nodules measuring 2 mm and 3 mm, respectively (Figure 1). These findings raised a high suspicion of metastatic disease.

Laboratory investigations revealed markedly elevated tumor markers, with alpha-fetoprotein (AFP) at 2800 ng/mL and carbohydrate antigen 19-9 (CA 19-9) at 3800 U/mL, while carcinoembryonic antigen (CEA) levels were within normal limits. These findings suggested a potential gastrointestinal or hepatic origin for the malignancy.

CA 125 was within normal limits, and PET/CT imaging showed multiple fluorodeoxyglucose (FDG)-avid lesions in the liver, the largest lesion measuring 8 x 6 cm in segment 4, along with non-FDG-avid retroperitoneal lymphadenopathy. A colonoscopy was performed and was negative. A mammogram was not performed, but the clinical breast exam was negative, and there was no evidence of breast tumor on either the CT or PET scan.

NGS was performed through Tempus; both liquid and tissue testing were done and revealed HER-2 amplification, with no other actionable targets.

Biopsy and immunohistochemistry findings

An initial liver biopsy yielded benign tissue, which was inconsistent with the imaging findings. Given the strong suspicion of carcinoma, a second liver biopsy was performed, revealing a poorly differentiated carcinoma with the following immunohistochemical profiles: positive markers, Oscar, AE1/AE3, SATB2, and HER2 (3+); negative markers, CK7, CK20, GATA3, CDX2, PAX8, TTF-1, and hormonal receptors; and a PD-L1 score of 5%.

NGS was obtained through Tempus; both liquid and tissue testing were done and was found to have HER-2 amplification, with no other actionable targets.

**Figure 2:**
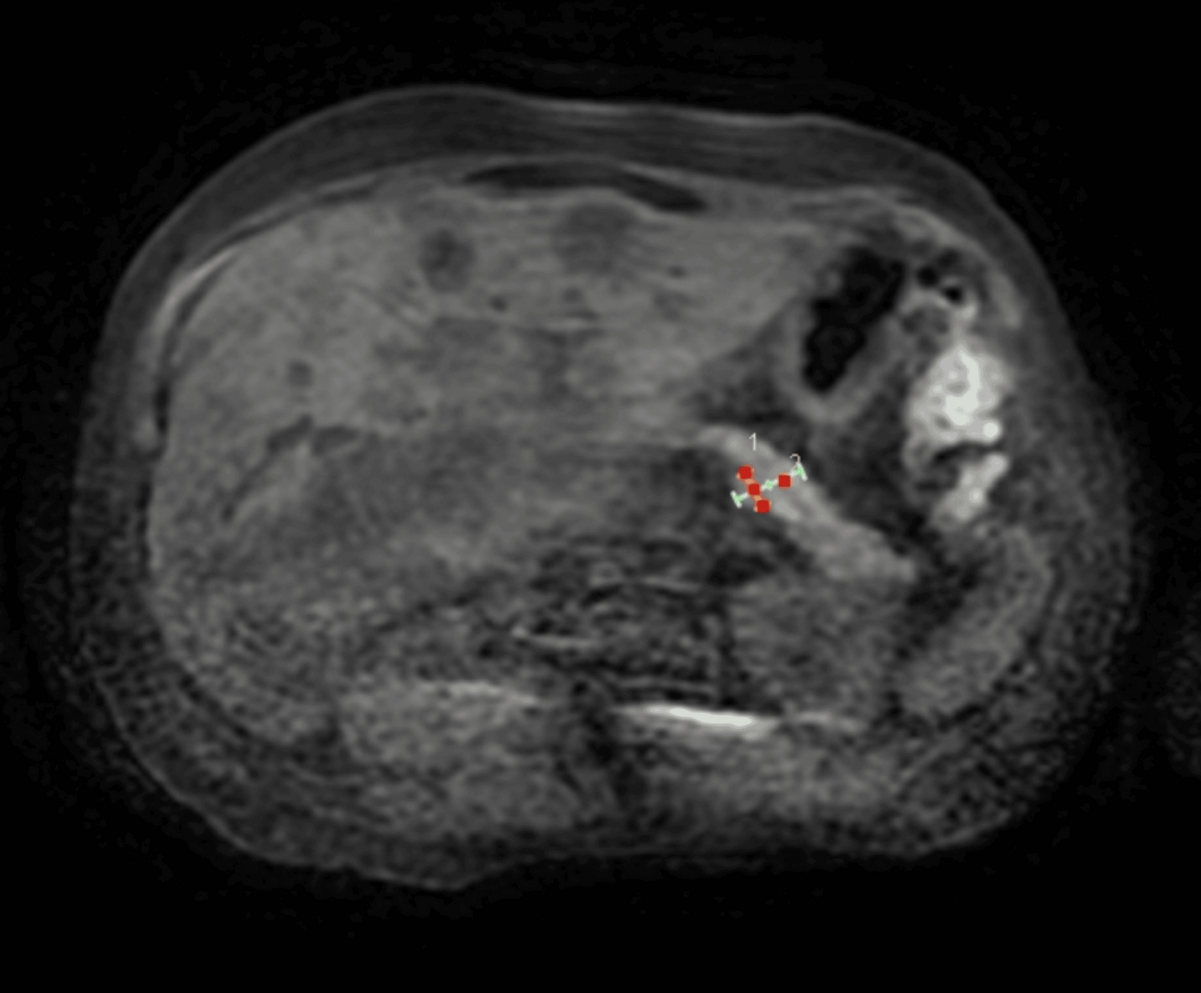
Abdominal and pelvic CT showing slight enhancement of the pancreatic duct at the junction of the body and tail.

These results suggested a HER2-positive carcinoma, although the precise origin remained undetermined. A multidisciplinary tumor board reviewed the findings and proposed a gastrointestinal origin based on the immunohistochemical and clinical presentation. Radiologic imaging revealed a slight enhancement of the pancreatic duct at the junction of the body and tail, prompting a recommendation for endoscopic retrograde cholangiopancreatography (ERCP) to further evaluate this area (Figure 2).

Treatment and clinical course

Given the patient’s extensive liver metastases, dramatic weight loss, significant pain, and overall frailty, the tumor board prioritized initiating systemic chemotherapy over additional diagnostic procedures, such as ERCP. The patient began treatment with carboplatin and gemcitabine, a regimen selected for its broad efficacy in gastrointestinal malignancies and tolerability in frail patients. HER2-directed therapy was reserved as a second-line option for potential progression based on the NGS findings that confirmed HER2 amplification (3+).

Transition to palliative care

During the first cycle of chemotherapy, the patient experienced profound fatigue and a decline in her performance status. Recognizing the limited benefit of aggressive treatment and the patient's desire to prioritize quality of life, she transitioned to hospice care. This decision allowed her to spend meaningful time with her family while receiving supportive care to manage symptoms and ensure comfort.

## Discussion

CUP remains a formidable diagnostic and treatment challenge due to its elusive nature [2]. This case highlights the typical course of a patient presenting with metastatic disease of unknown primary, compounded by a poor performance status that limits extensive procedural diagnostics [4]. Elevated AFP and CA 19-9 initially suggested gastrointestinal or hepatic origins, yet imaging and immunohistochemistry failed to definitively localize the primary site [3][5].

Although the slight enhancement in the pancreatic duct at the body-tail junction on the CT scan suggested further evaluation with ERCP, her extensive tumor burden, poor differentiation on biopsy, and unavailability of local services necessitated prioritizing chemotherapy over further diagnostics. Multidisciplinary discussions balanced the risks of delayed treatment with the potential benefits of additional procedures like ERCP [1][5].

The advent of NGS has significantly advanced oncology by enabling targeted therapies. In this case, HER2 amplification (3+) provided an opportunity to consider HER2-directed therapy, such as trastuzumab deruxtecan, approved for HER2-positive solid tumors [6]. Given the extensive liver metastases and frailty, the decision to proceed with carboplatin and gemcitabine, with a reduced carboplatin dose to mitigate toxicity, was considered appropriate. HER2-targeted therapy was planned as an adjunct in future treatments [5].

Cancer diagnosis and staging play critical roles in offering optimal treatment. Poorly differentiated tumors with unknown origins present unique challenges in achieving tailored therapies. This case underscores the importance of diagnostic testing and multidisciplinary discussions to optimize treatment plans for CUP.

## Conclusions

Carcinoma of unknown primary (CUP) remains a formidable challenge in oncology, characterized by its diverse clinical presentations, poor histologic differentiation, and the frequent inability to identify the primary tumor site despite extensive diagnostic efforts. These complexities necessitate a systematic and multidisciplinary approach to diagnosis and treatment, where a delicate balance must be struck between comprehensive diagnostic evaluations and the urgency of initiating therapy. In advanced cases with high tumor burden, timely therapeutic interventions take precedence, often requiring clinicians to forgo exhaustive investigations in favor of stabilizing the patient and addressing immediate concerns. The advent of NGS has revolutionized the management of CUP, offering a transformative opportunity to identify actionable molecular targets, such as HER2 amplification. This breakthrough enables the application of personalized therapies that can significantly improve outcomes, even in cases where traditional diagnostic pathways fail. Multidisciplinary collaboration is integral to CUP management, with tumor board discussions playing a crucial role in balancing diagnostic exploration with therapeutic planning. This collaborative framework ensures that treatment strategies are tailored to the patient’s clinical profile, particularly in frail or high-risk individuals, where decisions must weigh the benefits of broad-spectrum chemotherapy against the potential of targeted therapies. Timely treatment initiation is paramount in CUP, especially for patients with extensive metastatic disease and compromised functional status. Broad-spectrum chemotherapy often serves as the first-line approach to address aggressive disease, while molecular insights from NGS facilitate the integration of targeted therapies in subsequent treatment phases. This case emphasizes the pressing need for continued research to improve our understanding of CUP, enhance diagnostic methodologies, and refine therapeutic strategies. Such advancements are vital for improving patient outcomes and integrating emerging treatments into standard clinical practice. What sets this case apart is the inability to identify a primary tumor despite comprehensive diagnostic efforts, including multiple tumor markers, advanced imaging, and repeated biopsies, which ultimately revealed poor tumor differentiation. However, the application of molecular testing highlights a promising paradigm shift in CUP management. By identifying actionable targets such as HER2 amplification, even CUP patients can benefit from personalized and targeted therapies, offering new hope and paving the way for more effective treatment strategies in this challenging clinical entity.
